# Letter to the Editor: comment on “Arthroscopic suspension fixation for acute posterior cruciate ligament avulsion fractures in adolescents: a retrospective cohort study”

**DOI:** 10.1097/JS9.0000000000003569

**Published:** 2025-10-13

**Authors:** Guandong Chen, Yibing Chen, Weiguo Xu

**Affiliations:** a Academy of Medical Engineering and Translational Medicine, Tianjin University, Tianjin, China; b Department of Neurological Surgery, UCSF Medical Center, San Francisco, California, USA; c Department of Orthopedics, Cangzhou Central Hospital, Cangzhou, Hebei, China; d Tianjin Hospital, Tianjin University, Tianjin, China; e Queen Mary School, Nanchang University, Nanchang, Jiangxi, China

*Dear editor*

I have carefully reviewed the article by Chen *et al* entitled “Arthroscopic Suspension Fixation for Acute Posterior Cruciate Ligament Avulsion Fractures in Adolescents: A Retrospective Cohort Study.” This study focuses on a rare but clinically significant injury, and the authors should be commended for collecting adolescent cases and exploring the feasibility of a minimally invasive suspensory fixation technique. Nevertheless, from the perspectives of biomechanics and evidence-based medicine, several aspects of this work warrant further discussion.

In accordance with current reporting standards, this Letter complies with the TITAN 2025 guidelines**^[1]^**, which emphasize transparency, data integrity, human oversight, and the responsible use of artificial intelligence in surgical research and publication. The detailed information of the TITAN 2025 Guideline Checklist is provided in Supplemental Digital Content Table S1, available at http://links.lww.com/JS9/F337.

## Clinical relevance and study question

This topic is undoubtedly of considerable importance: posterior cruciate ligament tibial avulsion fracture (PCLTAF) in adolescents is extremely rare, and its optimal treatment strategy remains controversial. Most previous reports are limited to case studies or small case series. Although the present work represents a single-arm retrospective cohort study (Level IV evidence), it still carries potential clinical value. The authors appropriately focused on physeal safety, fracture healing, and functional recovery, which are all clinically relevant. However, the study did not reference or adopt any established classification systems based on radiographs or CT/MRI (such as the classic Meyers–McKeever classification**^[2]^**), nor did it provide classification-based statistical analyses or outcome stratification. Instead, adult fixation strategies were directly applied to the adolescent population. More critically, the study does not address a central long-term concern: can suspensory fixation reduce the risk of premature physeal closure or deformity? And compared with alternative methods – such as plate fixation, anchor fixation, screw fixation, suture fixation, or conservative treatment – does it offer a clear advantage? The authors proposed that suspensory fixation may promote healing through “micromotion” and cited Frost’s bone physiology theory**^[3]^**. While this hypothesis is thought-provoking, it lacks clinical or animal experimental validation. Prior comparative studies**^[4]^** have indicated that suture bridge and screw fixation may provide greater mechanical stability under cyclic loading. In the absence of direct comparative data, concluding that suspensory fixation is “superior” to screw fixation appears to be an overextension and limits its value for clinical decision-making.

## Outcomes and statistical issues

In Table 2, the notation “p = 0.000” is inappropriate and should be reported as “p<0.001.” Moreover, although the authors referred to the thresholds of the minimal clinically important difference and the patient acceptable symptom state, they mainly presented mean values, which may obscure individual variability. In addition, two patients (7%) who were lost to follow-up were directly excluded, and no intention-to-treat analysis was applied**^[5]^**. Neither statistical power nor effect size estimates were provided. The reported improvements in Lysholm and International Knee Documentation Committee (IKDC) scores (greater than 70 points) without major complications, while impressive, raise concerns regarding potential scoring bias, ceiling effects, or an overreliance on subjective outcome measures**^[6]^**. Objective stability assessments, such as KT-1000/2000 arthrometry or stress radiography, which are already considered standard practice in current studies**^[7]^**, should have been incorporated. Furthermore, although a mean follow-up of 30 months reflects mid-term outcomes, it is insufficient to exclude late degenerative changes or delayed growth disturbances. Therefore, the study conclusions should be interpreted with greater caution, with explicit acknowledgment that the long-term safety of this technique prior to skeletal maturity remains unproven.

## Literature contextualization

Although the authors cited some studies on screw and suture fixation, the discussion did not sufficiently situate the present work within the context of recent systematic reviews, nor did it address the ongoing academic debate regarding surgical displacement thresholds. There was a lack of discussion on recently reported pediatric case series**^[8,9]^**. The study results were not rigorously compared with prior reports of open reduction or arthroscopic fixation, and the fact that suture anchors or screw fixation have also been associated with favorable functional outcomes was not emphasized**^[10,11]^**. A more comprehensive comparative discussion would have substantially enhanced the scholarly contribution of this paper. Furthermore, while the perspectives on artificial intelligence and biomaterials are forward-looking, their relevance to the present study is limited, which detracts from the overall focus.

## Limitations disclosure

This study was a single-center retrospective analysis without a control group, which limits the strength of the evidence. The mean follow-up of approximately 30 months reflects only mid-term outcomes and is insufficient to evaluate long-term physeal consequences; late growth disturbances or degenerative changes cannot be entirely excluded. Case selection may have favored patients with better prognoses, introducing a degree of selection bias. In addition, knee stability was assessed primarily through subjective scores and physical examination, without the use of objective quantitative tools. Future research should consider incorporating classification systems based on CT and MRI to more accurately evaluate physeal risks in adolescents, and such approaches should be validated in prospective, multicenter studies with larger cohorts and extended follow-up to enhance the reliability and generalizability of the findings.

## Conclusion

This study provides valuable preliminary data in the field of adolescent PCLTAF and suggests that arthroscopic suspensory fixation may be a feasible option. However, the absence of objective stability measurements, the insufficient assessment of physeal safety, and the lack of comparative evidence substantially weaken the reliability of the conclusions. Future research should prioritize multicenter, prospective controlled trials that incorporate standardized biomechanical and functional endpoints, with extended follow-up until physeal closure and return to sports. Only through such rigorous evaluation can the true efficacy and safety of this technique be confirmed.

## Supplementary Material

## Data Availability

Not applicable.
